# Traumatic ulcerative granuloma with stromal eosinophilia — clinical case report, literature review, and differential diagnosis

**DOI:** 10.1186/s12957-019-1736-z

**Published:** 2019-11-09

**Authors:** Benito Benitez, Julia Mülli, Alexandar Tzankov, Christoph Kunz

**Affiliations:** 1 Department of Oral and Craniomaxillofacial Surgery, University Hospital Basel, Spitalstrasse 21, CH-4031 Basel, Switzerland; 2 Department of Histopathology, Institute of Medical Genetics and Pathology, University Hospital Basel, Schönbeinstrasse 40, 4031 Basel, Switzerland

**Keywords:** Traumatic ulcerative granuloma with stromal eosinophilia, TUGSE, TUGSE lesions, Riga-Fede disease

## Abstract

**Background:**

Traumatic ulcerative granuloma with stromal eosinophilia (TUGSE) is a rare self-limiting condition of the oral mucosa. The lesion manifests as an isolated ulcer that can be either asymptomatic or associated with mild to severe pain, and in most cases, it affects the tongue. TUGSE lesions may mimic malignancy such as squamous cell carcinoma, CD30 positive lymphoproliferative disorder, or infectious diseases such as primary syphilis, tuberculosis, or Epstein-Barr virus mucocutaneous ulcer. Histologically dominating cells are lymphocytes, histiocytes, and eosinophils.

**Case presentation:**

We describe a TUGSE case of a patient with a solitary ulcer on the lower left retromolar buccal plane. Upon presentation, the patient reported a swelling on the buccal mucosa of the left lower jaw since 1 year with rapid growth over the last days and mild pain while chewing. The diameter of the intraoral lesion on the lower left retromolar buccal plane was approximately 4 × 3 cm; the lesion presented as indurated base with a central superficial ulceration of 2 × 1 cm, indicative for a malignant process. Histologically, the ulceration showed an expanding, infiltrative, and vaguely granulomatous morphology, involving the superficial mucosa and the fatty tissue, and extended between the deep striated muscle fibers. The lesion was rich in lymphocytes, histiocytes, and eosionophils intermingled with activated T-blasts without phenotypic abnormalities. TUGSE was then diagnosed based on the phenotype (especially the lacking expression of CD30, the retained T-cell phenotype, and the absence of Epstein-Barr virus), the clinical presentation, and the morphology. Twenty-six months after diagnosis, no recurrence of the ulceration was seen.

**Conclusions:**

As TUGSE may mimic malignancy or infectious diseases, biopsy is mandatory and should be combined with thorough clinical examination. A screening for infectious diseases (mainly syphilis, Epstein-Barr virus, and HIV infections) must be performed routinely. In most cases, the lesions resolve spontaneously, obviating the need of further actions other than clinical follow-up. The pathogenesis of TUGSE lesions is still under debate, although local traumatic events and a locotypic immune response have been suggested to be major contributing factors.

## Background

Traumatic ulcerative granuloma with stromal eosinophilia (TUGSE) is a rare, benign, and self-limiting lesion of the oral mucosa. The pathogenesis remains uncertain. Although trauma seems to affect the development of TUGSE, the majority of cases present without evident trauma [1–3]. The tongue is the most commonly affected location, although other areas may also be involved, including the buccal and vestibular mucosa, palatal mucosa, retromolar area, gingiva, and the floor of the mouth [4]. There are two age peaks of incidence in TUGSE. The incidence of TUGSE peaks during the first 2 years of life, primarily in connection with teething, and between the fifth and seventh decade [4–6]. Men and women are nearly equally affected, with a minor female predominance [1, 2].

Clinically, TUGSE often manifests as an ulcer with elevated and indurated margins as well as a yellowish fibrinous base. As it grows fast, it is often assumed to represent a malignant process, such as a squamous cell carcinoma (SCC), a CD30 positive lymphoproliferative disorder (LPD), or an infectious disease, such as primary syphilis, tuberculosis, or Epstein-Barr virus (EBV)-associated mucocutaneous ulcer [5]. TUGSE can either present asymptomatically or are associated with mild to severe pain [4, 5]. The definitive diagnosis should be established by combined histological and clinical results. Histologically, the ulcerative lesion is characterized by a vaguely granulomatous, sometimes jigsaw-like appearance with a diffuse polymorphic inflammatory infiltrate of histiocytes, predominantly activated T-lymphocytes, and eosinophils, and it often extends into the submucosa, deeper muscle fibers, and salivary glands [2, 3, 5]. The lesion usually regresses spontaneously or after removal of possible triggers for microtrauma (e.g., artificial denture), within weeks to months. In a few cases, the healing process may take up to 1 year [3, 4]. Elovic reported that a delayed healing of oral TUGSE lesions can be associated with the lack of significant synthesis of transforming growth factor (TGF)-α and TGF-β by eosinophils infiltrating the lesions [7].

Here, we aim to report a case of TUGSE located on the lower left retromolar buccal plane, to review clinical and histopathological features, and to point out some differential diagnoses and clinical work-up of this entity.

TUGSE was first clinically described by Riga in 1881 and called the Riga-Disease. In 1890, Fede [8] was the first to histologically describe the lesion based on samples of two infants aged 8 and 10 months. In infants, the eosinophilic ulcer is thus referred to as Riga-Fede disease. The lesions are usually located sublingually or on the ventral side of the anterior tongue and are provoked by trauma from natal or neonatal teeth or newly erupted primary teeth [8, 9]. The first reports about those ulcerations localized on the oral mucosa were published between the 1950s and 1960s. The first case describing an analogous lesion in adults was published in 1956 by Popoff [10]. A few years later, Shapiro and Juhlin described this lesion as a separate independent entity [11]. Different terms have been used in the literature to describe this phenomenon ever since. It was called traumatic granuloma of the tongue [12], traumatic eosinophilic granuloma (TEG) [13], eosinophilic ulcer of the oral mucosa [5], eosinophilic ulcer of the tongue [11], and in 1983, Elzay named the disease traumatic ulcerative granuloma with stromal eosinophilia (TUGSE) [6]. Finally, Elzay proposed to include Riga-Fede disease as well as TEG into one entity (since they share obvious clinical and histological characteristics), and to use the term TUGSE [6].

## Case presentation

We detail the case of a 48-year-old man, never-smoker and non-drinker, who was referred to the Clinic of Oral and Craniomaxillofacial Surgery at the University Hospital Basel, Switzerland, by his dentist, for further evaluation of a solitary ulcer on the lower left retromolar buccal plane. The patient reported swelling of the left cheek since 5 days, mild pain while chewing, and a swelling on the buccal mucosa related to the left lower jaw since about 1 year. Fever, night sweat, and weight loss were denied. Due to his obsessive-compulsive disorder, the patient has been under psychiatric medication treatment in the past (*Deroxat®*, paroxetine, selective serotonin reuptake inhibitor (SSRI)), but at the point of consultation, he did not take any medication. The patient reported on his allergy to penicillin, with erythematous rash episodes in the past. The data set includes the medical record of our TUGSE case including the corresponding photo material. No risk for third parties existed. Informed consent was obtained from the patient for case description and photo material.

**Fig. 1:**
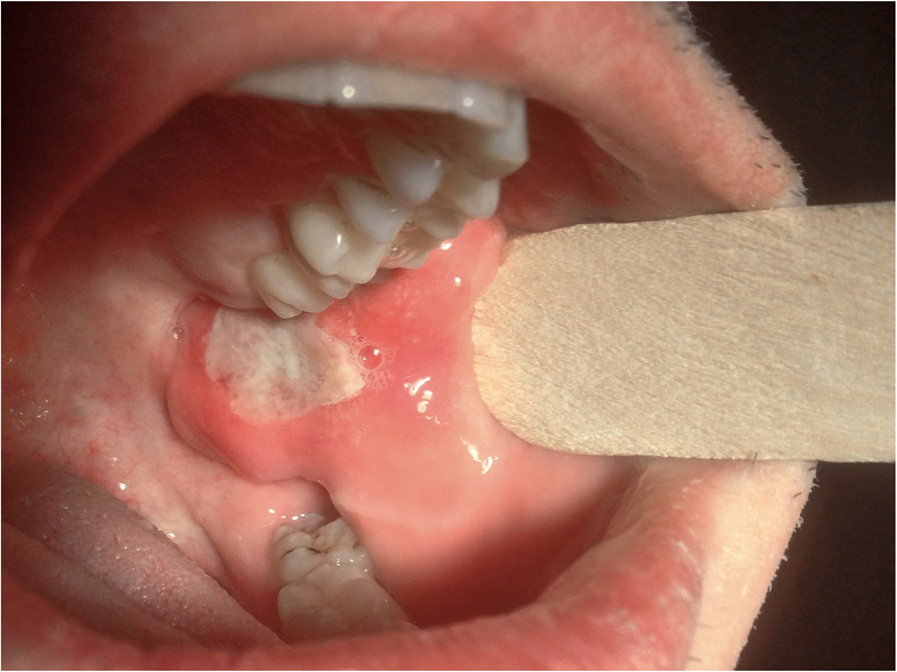
Clinical presentation of an oral TUGSE lesion on the lower left retromolar buccal mucosa in a 48-year-old patient

Extraoral examination showed a slight swelling of the cheek on the left side without redness. Palpation of the cervical lymph nodes revealed a slightly enlarged submandibular lymph node at level Ib on the left side, with a 2 × 1.5 cm diameter. Palpation of the immovable lymph node was painful. The nuchal, the axillary, the cubital, and the inguinal lymph nodes were not swollen. The diameter of the intraoral lesion on the lower left retromolar buccal plane was approximately 4 × 3 cm; the lesion presented as indurated base with a central superficial ulceration of 2 × 1 cm (Fig. 1). The rapid growth of the ulcer over the last 5 days was potentially indicative for a malignant process, wherefore we performed an incisional biopsy to obtain histopathological diagnosis after we had obtained informed consent from the patient. Tests for EBV, lues, HIV, hepatitis B, and hepatits C turned out to be negative. Nine days after the biopsy, the lesion showed a tendency towards regression.

**Fig. 2:**
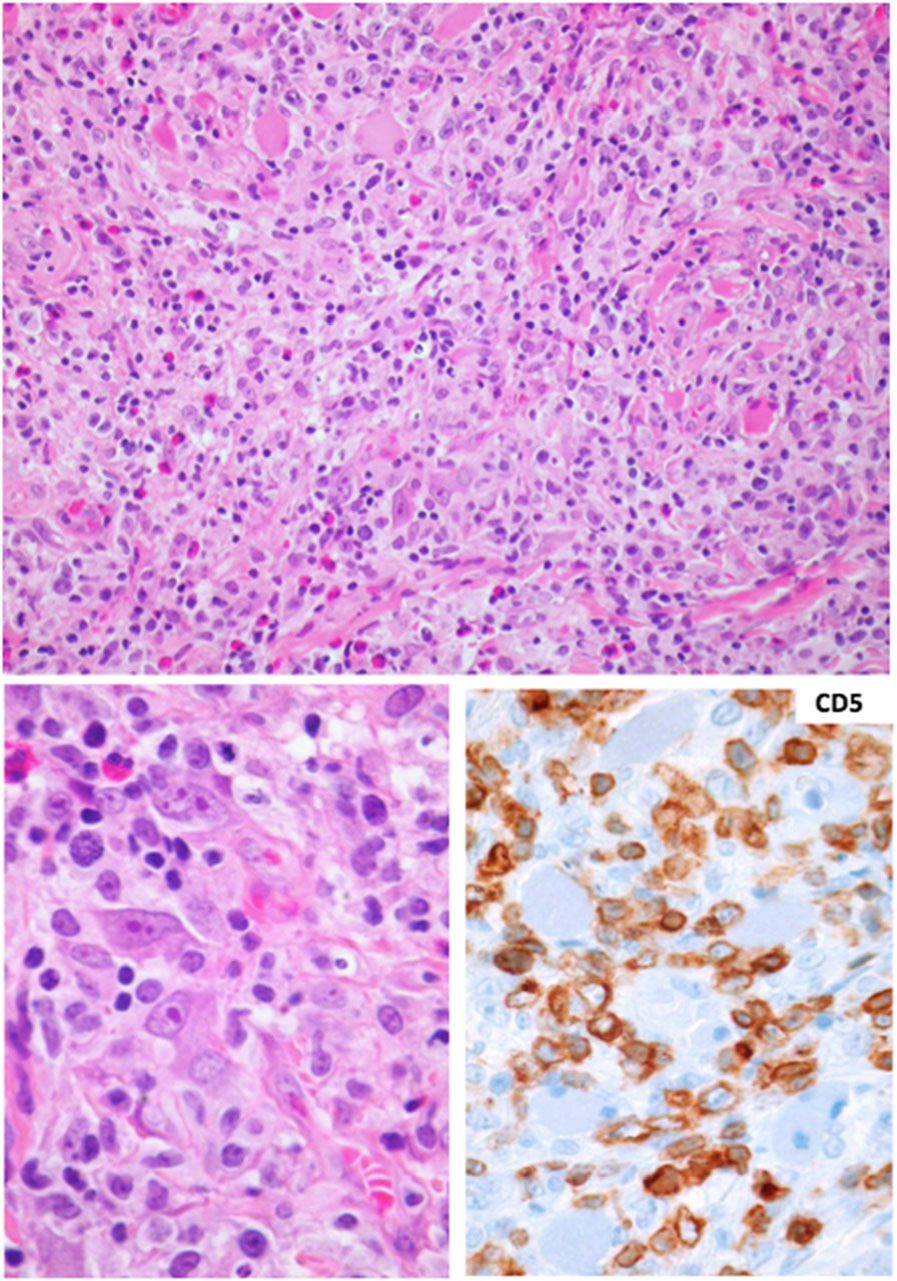
Jigsaw-like destructive infiltration of the submucosa by a mixture of pale appearing histiocytes, some cytologically atypical lymphocytes, and eosinophils; overview (H&E staining, × 100). Bottom left: detail magnification with centroblastoid and immunoblastoid large cells (H&E staining, × 360). Bottom right: positivity for CD5 of the smaller and larger lymphocytes in the lesion (immunoperoxidase staining, × 240)

Histologically, the ulceration showed an expanding, infiltrative, and vaguely granulomatous morphology, involving the superficial mucosa and the fatty tissue, and extended between the deep striated muscle fibers. The lesion was rich in lymphocytes, histiocytes, and eosionophils intermingled with activated T-blasts without phenotypic abnormalities (co-expressing CD2, CD3, CD5, and CD7, and lacking CD30) (Fig. 2). Although TUGSE was already presumed at that point, further molecular analyses were performed due to the large size of the lesion, its destructive and deep-penetrating nature, and the presence of large lymphoid forms with eosinophilic nucleoli. Deoxyribonucleic acid (DNA) was extracted from the mucosal biopsy. Using multiplex polymerase chain reaction and high-resolution fragment length analysis of the DNA, B-, and T-cell clonality as well as chromosomal translocations t(11;14) and t(14;18) were assessed [14]. A biclonal T-cell receptor rearrangement of type V-I and V-III was detected, but there was neither evidence of clonal B-cells nor of the translocations t(11;14) and t(14;18). TUGSE was then diagnosed on the basis of the phenotype (especially the lacking expression of CD30, the retained T-cell phenotype and the absence of EBV), the clinical presentation, and the morphology.

**Fig. 3:**
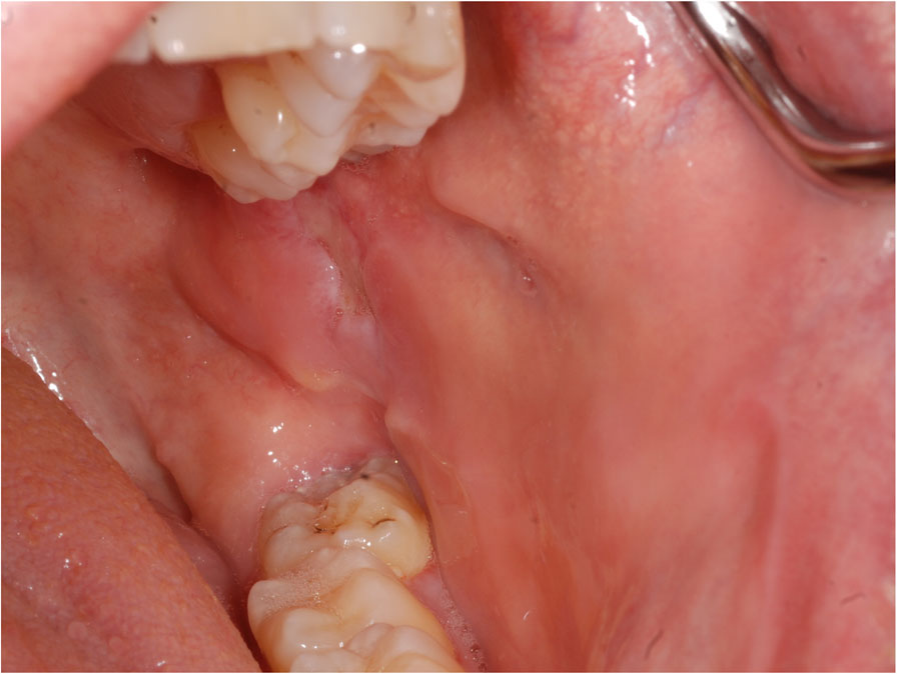
Remission of the ulcer 9 days after biopsy

**Fig. 4:**
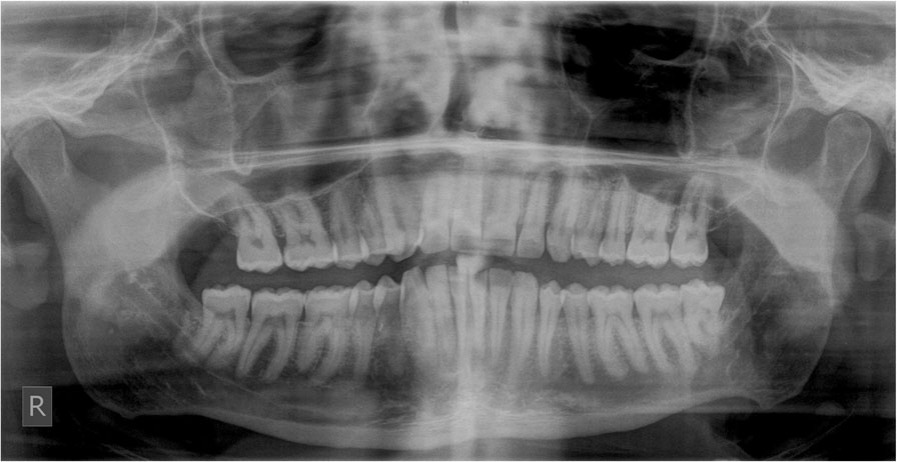
The orthopantomography (OPG) of our patient revealed no significant radiological findings

**Fig. 5:**
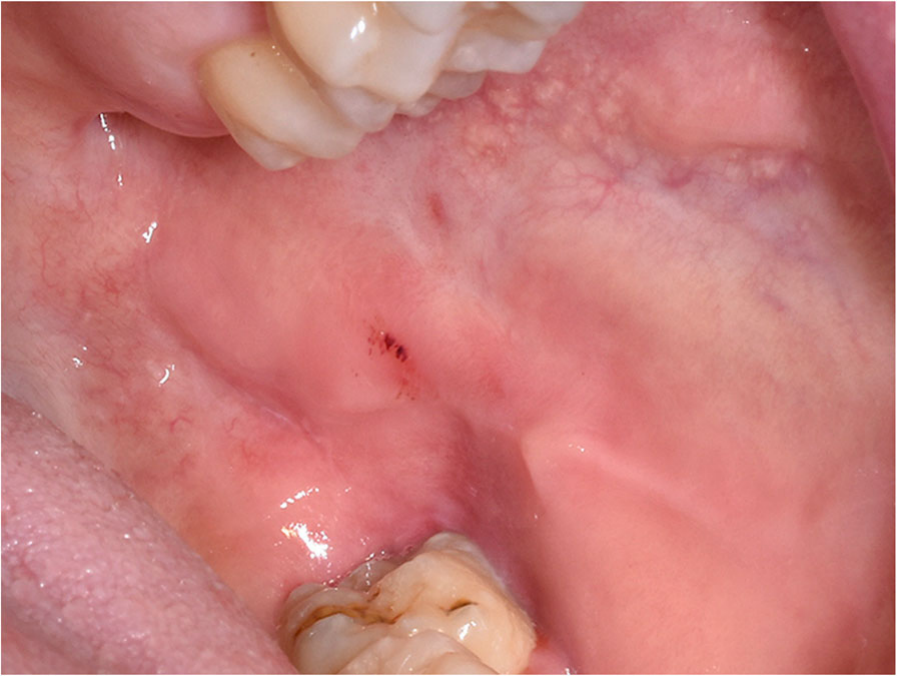
Twenty-six months after diagnosis the mucosa still shows signs of microtrauma with punctate hemorrhage, but the ulceration did not recur

Nine days after biopsy, the patient was pain-free and the ulceration showed significant regression (Fig. 3). We performed a standard orthopanthomography (Fig. 4), which revealed no other pathological finding than the clinical evident buccal angulation of tooth 38 which might have caused the microtrauma of the ulceration. We therefore recommended the prophylactic extraction of the respective tooth. However, the patient decided against it. We saw the patient in a regular follow-up program, quarterly within the first half year, every 6 months within the first year, and annually for 2 years. Twenty-six months after diagnosis, no recurrence of the ulceration was seen, even though the mucosa still showed signs of microtrauma with punctate hemorrhage (Fig. 5).

## Discussion

TUGSE is a rare but benign entity that can easily be mistaken for SCC, CD30 positive LPD, or infectious diseases, such as primary syphilis or EBV-associated mucocutaneous ulcer (Table 1) [4, 13, 15, 16]. With clinical inspection alone, a malignant process cannot be excluded, wherefore a biopsy is always mandatory. Screening for the aforementioned infectious diseases is recommended. The typical histological findings are vaguely granulomatous tissue changes, sometimes jigsaw-like appearance. The lesion presents a diffuse polymorphic inflammatory infiltrate, predominately consisting of histiocytes, activated and predominantly T-lymphocytes, and of eosinophils and histiocytes. The ulceration is often extending into the submucosa, deeper muscle fibers, and salivary glands. The pathogenesis of this lesion is still under debate, although a local traumatic event has been suggested to be a major contributing factor, as it also became apparent in our case. The traumatic origin in humans was first proposed after a series of cases analyzed by Fede in 1890 [8]. Yet, trauma can only be identified in less than 50% of cases [5, 6]. By means of experimental rat studies, Bhaskar and Lilly [12] observed TUGSE-like lesions on the animals’ tongues after repeated injury of the respective areas. They concluded that the term *traumatic granuloma* is appropriate because the lesion is “essentially reactive and a result of trauma” [12].

**Table 1:** Differential Diagnosis of TUGSE

Parameter	Traumatic ulcerative granuloma with stromal eosinophilia (TUGSE)	Squamous cell carcinoma (SCC)	CD30 positive lymphoprolipherative disorder (LPD)	Lues (syn. Syphilis)	Epstein-Barr virus (EBV) mucocutaneous ulcer	Aphthous stomatitis
Location in the oral cavity	Tongue, buccal and vestibular mucosa, palatal mucosa, retromolar area, gingiva, floor of the mouth	Bottom of the oral cavity, lateral side and tip of the tongue, lower lip, retromolar area	Rarely in the oral cavity, often recurring lesions	Lips, tongue, pharynx	Oropharyngeal mucosa	Non-keratinizing epithelial surfaces in the mouth (labial and buccal mucosa, soft palate, lower side of the tongue); in patients with Severe aphthous stomatitis, the keratinized mucosa can also be affected
Etiopathogenesis	Unknown, causal connection with repeated trauma	Causal connection with chronic tobacco and alcohol consumption and poor oral hygiene	Typically in patients with an impaired immune system	Most commonly spread through unprotected (oral) sexual intercourse or congenital infection	Associated with immunosuppression	Stress, trauma, hormonal fluctuations, allergic reaction (certain foods, drinks, toothpastes and mouth rinses)
Peak age	Two peaks of life incidence: one during the first two years of life and another between the 5th and 7th decade	Most commonly after the 5th decade of life (men more frequently affected than women)	Elderly (> 75 years)	Most commonly between the 3rd and the 4th decade of life (men more frequently affected than women)	Primarily elderly (> 75 y), any age	Any age but the frequency increases later in life
Clinical features	Ulcer with elevated and indurated margins and yellowish fibrinous base	Endophytic growth, nodose and shallow ulcer with elevated margins, often covered by a non-strippable reddish, whitish or mixed focus growing infiltrative and destructive	Nodules or ulceration, indolent clinical behavior, primary cutaneous manifestation possible	Stage I (primary syphilis): after an incubation period of 2–3 weeks, the papule at the portal of entry converts into a indolent and superficial ulcers with indurated margins (also called primary chancre) multifocal, aphthous enanthema	Sharply circumscribed indolent ulceration	Nonspecific shallow round or oval painful ulcer, covered by a grayish-white fibrin pseudomembrane with a sharply defined erythematous border
Histopathology	Granulomatous tissue with a dense, diffuse, polymorphic, inflammatory infiltrate predominately of eosinophils and histiocytes, often extending into the submucosa, deeper muscle fibers and salivary glands	Epithelial differentiated structures with cornification (hyperkeratosis, parakeratosis, horn beads and multiple dyskeratotic keratinocytes) and peritumoral inflammatory reaction composed of a mixed cellular infiltrate, which is rich in plasma cells	Infiltrate of atypical lymphoid cells admixed with eosinophils involving the oral epithelium and the deep soft tissues	Significant plasmacytosis, plasmacell phlebitis, vague granulomas, detectable spirochetes (Warthin-Starry, immunohistochemistry)	Polymorphous infiltrate with inflammatory cells and atypical large B-cells blasts often with Hodgkin/Reed-Sternberg (HRS) cell-like morphology, sharply demarcated towards deeper structures (assessible only on excisional biopsies)	Nonspecific ulcer, inflammatory cells, predominately T-cells, with high local levels of TNF-α
Immunohistochemistry	Mixture of phenotypically regular T-lymphocytes, occasionally CD30+	CK5/6+, CK19+, p63+, p40+	MUM1p+, MYC+, CD30+ T-cells with antigenic loss of T-cell markers	Detection of *Treponema pallidum* by the following tests: TPPA test (Treponema pallidum particle agglutination assay test), FTA-ABS test (fluorescent treponemal antibody absorption test)	EBER+ (EBV encoded small nuclear RNA), CD30+	Nonspecific

In the majority of the cases, the lesions heal spontaneously [9]. Except for the incisional biopsy for definitive diagnosis, only regular observation is required because the resolution of the lesions may take weeks up to several months, and in few cases up to 1 year [3, 4]. Application of topical corticosteroids, like triamcinolone acetonide ointment, had no additional benefit. Recurrence has been reported in some cases [6]. Because the integrative diagnosis of TUSGE is mostly based on exclusion of other, particularly malignant disorders, clinical follow-up should be performed in all cases, even after the lesions are completely removed.

In our case, the histologically dominant cells in the infiltrate were smaller lymphocytes, histiocytes, and eosionophils continuously streaked by T-lblasts. Degranulating eosinophils and toxic products or cytotoxic T-cells cause the typical mucosal degeneration of TUGSE lesions [3]. Interestingly, Elovic et al. [7] found that the expression of TGF-α or TGF-β1 in the eosinophils of TUGSE lesions was significantly decreased compared to the eosinophils in normal wounds. They suggested that the typical delayed healing in TUGSE lesions is associated with the lack of synthesis of TGF by eosinophils.

The immunohistochemical characteristic of TUGSE has been a matter of debate due to the unidentified origin of the large, atypical mononuclear cells. Authors have suggested their origin in macrophages (CD68 positive cells) [3, 13], dendritic cells (factor XIIIa positive cells) [3], and myofibroblasts (vimentin positive cells) [3]. Yet these large, atypical mononuclear cells (often CD30 positive) most likely originate from T-lymphocytes, as they often express T-cell markers or/and cytotoxic markers, and often display clonal T-cell receptor gene rearrangements, as in our case. They might play a role in the reparative phase of the lesion. In 1997, Ficarra et al. [13] for the first time described a case of TEG, in which CD30-positive cells in an ulcerated lesion could be evidenced. Subsequently, other reports revealed CD30-positive eosinophilic ulcers. CD30-positive large atypical cells can be observed in TUGSE lesions in a scattered or clustered manner [3, 5]. Therefore, these lesions were considered the oral counterpart of the spectrum of primary cutaneous CD30-positive LPDs by some authors. CD30 is commonly expressed on activated B- and T-cells and is a useful histological marker for a spectrum of LPDs, including Hodgkin lymphoma. Yet many non-neoplastic cutaneous disorders, such as atopic dermatitis, drug reactions, molluscum contagiosum, and scabies, can contain CD30 positive cells [1, 3]. The CD30 positivity of some TUGSE lesions is most probably a sign of an unspecific T- and/or B-cell activation, as suggested by Segura and Pujol [1], who considered eosinophilic ulcer of the oral mucosa to be a nonspecific locotypic reaction rather than a distinct entity.

The majority of the TUGSE cases available in the literature are published in oral and maxillofacial pathology journals [6, 7, 9, 12, 13]. In the dermatological literature, similar cases are only rarely described [5]. To recommend adequate treatment strategies, more cases and systematic analyses are needed, in order to draw attention to this entity, to understand the process of formation of TUGSE, and to uncover targetable pathogenetic pathways.

## Conclusions

As TUGSE may mimic malignancy or infectious diseases, biopsy is mandatory and should be combined with thorough clinical examination. A screening for infectious diseases (mainly syphilis, EBV, and HIV infections) must be performed routinely. In the majority of cases, the lesions resolve spontaneously, obviating the need of further actions other than clinical follow-up, which is advisable even in complete remission. The pathogenesis of TUGSE lesions is still under debate, although local traumatic events and a locotypic immune response have been suggested to be major contributing factors.

## Data Availability

The datasets used and/or analyzed during the current study are available from the corresponding author on reasonable request.

## Abbreviations

DNA: Deoxyribonucleic acid
EBV: Epstein-Barr virus
HIV: Human immunodeficiency virus
LPD: Lymphoproliferative disorder
PCR: Polymerase chain reaction
SCC: Squamous cell carcinoma
TEG: Traumatic eosinophilic granuloma
TGF: Transforming growth factor
TUGSE: Traumatic ulcerative granuloma with stromal eosinophilia
